# Quality of life ratings in dementia care – a cross-sectional study to identify factors associated with proxy-ratings

**DOI:** 10.1186/s12955-014-0177-1

**Published:** 2014-12-12

**Authors:** Johannes Gräske, Saskia Meyer, Karin Wolf-Ostermann

**Affiliations:** Department 11: Human and Health Sciences, University of Bremen, Grazer Str. 4, 28359 Bremen, Germany

## Abstract

**Objective:**

Quality of life (QoL) is one major outcome parameter in the care for people with dementia (PwD); however, their assessment is lacking a gold standard. The purpose of this study was to evaluate potential factors associated with nurse-rated quality of life of PwD in nursing homes in Berlin, Germany.

**Method:**

An explorative cross-sectional study was performed in five nursing homes to evaluate QoL. Nurses rated the QoL for all residents with dementia by completing two different standardised assessments (ADRQL, QUALIDEM). Potential associated factors were evaluated concerning resident and nurse related factors. A fixed-effects models of analysis of co-variance (ANCOVA) was used to analyse effects of assumed associated factors of the major outcome parameters ADRQL and QUALIDEM. Associated factors were severity of dementia (GDS), challenging behaviour (CMAI), and other characteristics. Regarding the nurses, burnout (MBI), satisfaction with life (SWLS), attitude (ADQ) and empathy toward residents (JSPE), as well as circumstances of the ratings and days worked in advance of the ratings were assessed.

**Results:**

In total, 133 PwD and 88 nurses were included. Overall, the ratings show moderate to high QoL in every subscale independent of the instrument used. Assumed confounders relevantly influenced 14 out of 17 ratings. Predominantly, residents’ challenging behaviour, nurses’ burnout and satisfaction with life as well as the circumstances of the ratings are significant and clinically relevant associated factors.

**Conclusion:**

Assessing QoL of PwD is acknowledged as a central component of health care and health care research. In later stages of dementia, proxy-reported information obtained from quality of life questionnaires is and will continue to be essential in this research. However, methodological issues that underline this research - matters of measurement and instrument validity - must receive more attention. Associated factors in proxy-ratings have to be routinely assessed in order to get more valid and comparable estimates.

## Introduction

Worldwide, the number of persons with dementia (PwD) is increasing [1]. Therefore, care of PwD is challenging and becomes a focal point of interest. Although most PwD are community-dwelling and cared for by relatives and friends, dementia is one major reason for relocation into a nursing home [2]. Various studies have aimed to improve the care provision in those settings. To evaluate the impact of an intervention, quality of life (QoL) is considered one of the main parameters; it includes objective and subjective aspects of PwD [3,4].

### Quality of life ratings

The evaluation of QoL of PwD is associated with various problems [5], especially because there is no ‘gold standard’ [6]. However, the evaluation of QoL is possible in self and/or proxy-ratings [7]. In addition, due to the subjective aspects of the concept of QoL, self-rating scales are considered the best way to evaluate QoL [8] but in longitudinal interventional studies it is unclear whether changes in QoL are caused by the progression of the dementia and associated disorders or actually by the interventional program. Moreover, in a population with severe dementia, PwD are frequently unable to understand the questions or even remember the situation about which they are asked [9]. Therefore, especially in a population with severe dementia, proxy-ratings (e.g., by nurses) are the method of choice and reduce the number of missing values [10]. Typically, dementia-specific QoL questionnaires show a sufficient reliability, e.g., inter-rater reliability [7]. However, associated factors reliability have yet to be investigated. In addition, proxy-ratings are associated with various unknown factors. This leads to a weak level of agreement between self- and proxy-ratings [10,11]. Few studies report predictors of a higher level of agreement between PwD and family members [12,13]. Studies investigating the ratings of PwD and nurses determined that being the responsible nurse [10] and staff age [11] are factors which improve the general level of agreement.

In various studies, effects of an intervention on proxy-rated QoL were found [14,15]. Other studies did not find convincing effects [16,17]. In these examples, the results are discussed only in light of the research questions. However, it is unclear if the ratings themselves are influenced by factors other than the intervention and it is unknown which factors might influence nurse-ratings basically.

A deeper insight into associated factors of proxy-ratings of QoL is required. The investigation of *‘factors that affect both patient and caregiver ratings […] of QoL*’ [18] is recommended. Those factors can be characteristics of nurses (e.g., burnout, attitude toward PwD) but also circumstances of the rating (e.g., at beginning of the shift, after holiday). To optimise the interpretation of interventional results, a valid QoL evaluation with individuals who have a severe level of dementia, is necessary. Therefore, the present study aims to investigate:

- variability of nurses rated QoL of PwD in institutional long-term care facilities in Germany
- PwD and nurse related factors associated with nurses rated QoL

## Method

### Sample and setting

Five nursing homes with ten wards for PwD participated in the study. All residents with a medical diagnosis of dementia and all nurses, either registered nurses or nursing assistances, working predominantly in a participating ward were included in the study.

### Procedure

In an explorative cross-sectional study, written study information was sent to heads of ten randomly selected nursing homes in Berlin, Germany, with the request to participate in the study. After four weeks, all nursing homes were contacted by phone. Primary nurses of each resident provided socio-demographic (age, sex) and further resident related characteristics (e.g., severity of dementia, need-based behaviour) on a written questionnaire. Afterwards each nurse received a written standardised questionnaire to rate the QoL of each resident in their ward. In addition, nurses provided information on their own socio-demographic characteristics (e.g., sex, age, time of being in the ward, being the responsible nurse). Additionally, nurses rated their own attitude towards PwD, empathy, satisfaction with life, burnout, days worked in advance of the ratings, and circumstances (e.g., before starting the shift) of the ratings.

### Quality of life measurements

The main focus within this article is measuring quality of life of PwD in nursing homes. To avoid instrument-related effects, two different proxy-rated QoL-instruments were applied as primary measurements. However, only two proxy-rated quality of life instruments, focusing on institutionalised people with all stages of dementia, the ADRQL and the QUALIDEM, are available in a validated German version.

### Alzheimer’s Disease Related Quality of Life (ADRQL)

The Alzheimer’s Disease Related Quality of Life (ADRQL) [19] assessment consists of 47 items concerning observable behaviour within the last two weeks. Their occurrence can be ‘agreed’ or ‘disagreed’ upon. The authors defined an item-specific value to weight the ratings. A relative global score and five relative scores for subscales (‘social interaction’; ‘awareness of self’; ‘enjoyment of activities’; ‘feelings and mood’; ‘response to surroundings’) were calculated. Due to relative score calculations, an imputation of missing values is not necessary. A higher score (0-100) indicates higher QoL. The German version of the ADRQL shows sufficient to good reliability. The validity was proofed using discriminant and convergent, as well as construct, validity by confirmatory factor analysis [20].

### QUALIDEM

The QUALIDEM [21,22] is a 37-item instrument for PwD. It includes nine subscales (‘care relationship’; ‘positive affect’; ‘negative affect’; ‘restless tense behaviour’; ‘positive self-image’; ‘social relations’; ‘social isolation’; ‘feeling at home’; ‘having something to do’) for mild to moderate dementia. For people with severe dementia, the number of items is 18 and only six subscales (excluded are: ‘positive self-image’; ‘feeling at home’; ‘having something to do’) are calculated. Various behaviours of the past week were rated from 0 = ‘never’ to 3 = ‘frequently’ or vice versa. The expectation-maximisation algorithm was used to impute missing values. A higher sum score indicates higher QoL. The global QoL score was calculated by summing up all items, without any weighting. To increase comparability, scores of all subscales and the global scores were linearly adapted to a scale from 0-100 (per scale: Σitem_i_ × 100 / 3 × item_i_). The German QUALIDEM versions show a weak inter-rater reliability [23]. The validity was again proofed using discriminant and convergent, as well as construct, validity by confirmatory factor analysis [20].

### Measurements of associated factors

PwD-related measurements included the severity of dementia, using the Global Deterioration Scale (GDS) [24]. A higher stage indicates a more severe level of dementia. Challenging behaviour of residents was assessed by using the Cohen-Mansfield Agitation Inventory (CMAI) [25]. Responsible nurses rated 29 behaviours on a seven point Likert-scale (from ‘never’ to ‘a few times in an hour’). The analysis indicated whether aggressive behaviour, physically nonaggressive behaviour or verbally agitated behaviour occurred. Further nurse-related outcomes included burnout as evaluated by using the Maslach Burnout Inventory (MBI) [26]. A 25 item questionnaire with ratings from ‘never’ to ‘frequently’ form three subscales: ‘emotional exhaustion (EE)’, ‘depersonalization (D)’, and ‘personal accomplishment (PA)’. For each subscale mild, moderate or severe burden of nurses is assessed. The attitude towards PwD was evaluated by applying the Approach to Dementia Questionnaire (ADQ) [27]. Nurses had to rate 19 statements on a five-point Likert-scale from ‘strongly disagree’ to ‘strongly agree’. These ratings result in a global sum score (19-95 points) and the domains ‘person centeredness’ (11-55) and ‘hope’ (8-40). Higher scores indicate a more positive attitude towards PwD. The Jefferson Scale of Physician Empathy (JSPE) [28], a questionnaire with 20 statements each rated on a seven-point Likert-scale from ‘strongly disagree’ to ‘strongly agree’, was used to assess the empathy of nurses. A higher sum score (20-140 points) indicates higher empathy. The Satisfaction with Life Scale (SWLS) [29] was used to evaluate nurses’ satisfaction with life. Five aspects were rated on a 7-point Likert-scale (ranging from ‘extremely unsatisfied’ to ‘extremely satisfied’). A global sum score was calculated, higher scores (5-35) indicate higher satisfaction with life. Additionally, basic characteristics of residents (e.g., age, sex) and nurses (e.g., age, sex, being the responsible nurse, being a registered nurse) were surveyed.

All associated variables show good psychometric properties for applied versions [24-29].

### Statistical analysis

Basic characteristics of residents and nurses were described using descriptive statistics. Correlations among metric/ordinal variables were examined by Pearson’s and Spearman’s correlations. Fixed-effects models of analysis of co-variance (ANCOVA) were used to investigate effects of assumed categorical and continuous associated factors of the major outcome parameters ADRQL and QUALIDEM (total score and subscales for each). These models were adjusted for confounding factors such as severity of dementia (GDS; levels ≤4, 5, 6, 7), occurrence of at least one challenging behaviour (CMAI), residents’ sex, nurses’ sex, being the responsible nurse (yes/no), being a registered nurse (yes/no), burnout (‘MBI-EE’, ‘MBI-PA’, ‘MBI-D’), circumstances of ratings, time being on the ward (nurses), attitude (ADQ total score), empathy (JSPE), satisfaction with life (SWLS) and days worked in advanced of the ratings (nurses). Interactions between confounding variables were not modelled because of the small sample size. Statistical model assumptions of normal distribution and multicollinearity for variables were examined before conducting further analyses. Two-sided significance was set at p ≤ 0.05. Due to multiple testing, Bonferroni-correction was applied for ADRQL and QUALIDEM analyses (total score and subscales; α = 0.003). All statistical analyses were carried out using SPSS^®^ (v21.0).

The study was conducted in line with German law and the declaration of Helsinki of the World Medical Association. The Ethics Committee of the German Society of Nursing Sciences approved the study protocol.

## Results

In total, five nursing homes with ten wards participated in this study. For all eligible residents (n = 133) data was collected. The number of participating nurses was 88, this corresponds to a response rate of 86.6%. In Table 1, characteristics of residents and nurses are shown. No significant correlations between residents’ measurements appeared in the analyses and all measurements of possible confounding factors were included in the analyses of (co-)variance. Regarding nurses’ characteristics, the only significant correlations were found between ADQ total score and ADQ subscales ‘person centeredness’ (r = 0.870 [95% CI: 0.758; 0.982], p < 0.001) and ‘hope’ (r = 0.792 [95% CI: 0.653; 0.930], p < 0.001), all possible confounding factors were included in the analyses of (co-)variance.

**Table 1:** **Characteristics of study sample**

	**Residents (n = 133)**		
Age in years; mean (SD)	85.4 (7.2)		
Women in % (n)	81.4 (105)		
Time being in the ward in years; mean (SD)	2.6 (2.7)		
Severity of dementia (GDS) in % (n)			
≤4 (no to moderate cognitive decline)	1.5 (2)		
5 (moderately severe cognitive decline)	3.0 (4)		
6 (severe cognitive decline)	21.8 (29)		
7 (very severe cognitive decline)	73.7 (98)		
Challenging behavior (CMAI) in % (n)			
Physically nonaggressive behavior	51.1 (68)		
Verbally agitated behavior	37.6 (50)		
Aggressive behavior	21.1 (28)		
Minimum one challenging behavior	63.2 (84)		
	**Nurses (n = 88)**		
Age in years; mean (SD)	37.2 (10.3)		
Women in % (n)	88.1 (74)		
Profession in% (n)			
Nursing assistances	47.7 (42)		
Registered nurses	52.3 (46)		
Time of work experiences in years; mean (SD)	7.4 (7.4)		
Time on the ward in years; mean (SD)	2.5 (2.3)		
Attitude towards people with dementia (ADQ); mean (SD)			
Total (19-95)	72.1 (7.2)		
Person centeredness (11-55)	44.3 (4.7)		
Hope (8-40)	27.9 (3.8)		
Empathy (JSPE; 20-140; mean (SD))	83.9 (6.0)		
Burnout (MBI) in % (n)			
	Low burden	Moderate burden	Severe burden
Emotional exhaustion	17.0 (15)	37.5 (33)	25.0 (22)
Depersonalization	14.8 (13)	54.5 (48)	11.4 (10)
Personal accomplishment	26.1 (23)	42.0 (37)	13.6 (12)
Satisfaction with life (SWLS; 5-35; mean (SD))	24.4 (6.5)		

SD: standard deviation; GDS: Global Deterioration Scale; CMAI: Cohen-Mansfield Agitation Inventory; ADQ: Approach to Dementia Questionnaire; JSPE: Jefferson Scale of Physician Empathy; Scale of MBI: Maslach Burnout Inventory; SWLS: Satisfaction with Life Scale.

### Quality of life

A total of 1,482 QoL ratings for ADRQL and QUALIDEM (each) could be included in further analyses. On average for each resident QoL was rated 11 times, by using the ADQRL and QUALIDEM. Each nurse rated QoL16.8 times. Within these ratings there was 3.7% missing items for the ADRQL and 4.1% missing values for the QUALIDEM.

The circumstances of ratings are comparable between both instruments. Predominantly, ratings are done during the shift (ADRQL: 33.5%; QUALIDEM: 33.4%) or shortly afterwards (ADRQL: 29.4%; QUALIDEM: 29.2%). Rarely, QoL ratings are done in advance of a shift (ADRQL: 12.4%; QUALIDEM: 13.3%). All other ratings are done during leisure time. Before performing ADRQL and QUALIDEM ratings, nurses worked on average 3.8 (SD 2.5; min: 0; max: 6) days.

The average QoL scores are displayed in Table 2. All scores indicate a moderate to high QoL of residents. The ADRQL and QUALIDEM scores are comparable; however, the standard deviations (ADRQL: 17.5 up to 22.9; QUALDEM: 18.7 up to 29.9) indicate heterogeneous ratings.

**Table 2:** **Quality of life scores (ADRQL, QUALIDEM)**

**ADRQL**	**n = 1.482**
Total	69.9 (18.0)
Social interaction	75.9 (20.8)
Awareness of self	63.1 (22.8)
Enjoyment of activities	74.5 (22.9)
Feelings and mood	57.3 (17.5)
Response to surroundings	78.0 (20.4)
**QUALIDEM**	
Total	70.8 (22.1)
Care relationship	70.5 (22.9)
Positive affect	73.3 (25.9)
Negative affect	72.0 (25.9)
Restless tense behavior	55.1 (29.9)
Positive self-image*	78.7 (24.9)
Social relations	74.3 (21.9)
Social isolation	71.5 (21.9)
Feeling at home*	84.2 (18.1)
Having something to do*	54.5 (26.3)

Data represent means (standard deviation); *not for GDS = 7; for all scales: 0-100.

### Associated factors of the ADRQL

Tables 3 and 4 show the results of the ANCOVA-analyses for the ADRQL. Regarding the corrected alpha-level, all six models show significant (all ANCOVA, p < 0.003) results with a proportion of explained variance up to one quarter (R^2^ up to 0.266). Altogether, ten associated factors were identified. Variables associated with each of the five scores are: severity of dementia (GDS), nurses’ satisfaction with life (SWLS) (‘total score’, ‘social interaction’, ‘awareness of self’, ‘moods and feeling’, ‘enjoyment of activities’) and circumstances of ratings (‘total score’, ‘social interaction’, ‘awareness of self’, ‘moods and feeling’, ‘response to surroundings’). Three scores (‘total score’, ‘moods and feeling’, ‘response to surroundings’) are influenced by the occurrence of challenging behaviour (CMAI) and the time nurses are already in the ward. Higher burnout of nurses (‘total score’, ‘moods and feelings’) and the number of days nurses worked in advance of the ratings (‘awareness of self’, ‘response to surrounding’) influence two scores. The direction of influence is displayed in Table 4.

**Table 3:** **Factors associated with ADRQL-ratings**

**Dependent variable**	**p-value**	**R** ^**2**^	**Significant independent variable**
ADRQL**: Total score**	**p < 0.001**	0.244	Challenging behavior (CMAI)^★^
Non-significant independent variable: 3, 4, 5, 6, 8, 9, 12, 13, 15 (see underline)			Severity of dementia (GDS)^★★★^
			Burnout (MBI – EE)^★★^
			Circumstances of ratings^★★★^
			Time being on the ward (nurse)^★★^
			Satisfaction with life (SWLS)^★★★^
ADRQL**: Social interaction**	**p < 0.001**	0.170	Severity of Dementia (GDS)^★★^
Non-significant independent variable: 1, 3, 4, 5, 7, 8, 9, 11, 12, 13, 15 (see underline)			Registered nurse (yes/no)^★★^
			Circumstances of ratings^★★^
			Satisfaction with life (SWLS)^★^
ADRQL**: Awareness of self**	**p < 0.001**	0.227	Severity of dementia (GDS)^★★★^
Non-significant independent variable: 1, 3, 4, 5, 6, 7, 8, 9, 11, 12, 13, (see underline)			Circumstances of ratings^★★^
			Satisfaction with life (SWLS)^★★^
			Days worked in advance of the ratings^★^
ADRQL: **Moods and feelings**	**p < 0.001**	0.266	Challenging behavior (CMAI)^★★^
Non-significant independent variable: 3, 4, 5, 6, 9, 12, 13, 15 (see underline)			Severity of dementia (GDS)^★^
			Burnout (MBI – EE)^★★^
			Burnout (MBI – PA)^★★^
			Circumstances of ratings^★★★^
			Time being on the ward (nurse)^★★★^
			Satisfaction with life (SWLS)^★★★^
ADRQL: **Enjoyment of activities**	**p = 0.002**	0.140	Severity of dementia (GDS)^★★★^
Non-significant independent variable: 1, 3, 4, 5, 6, 7, 8, 9, 10, 11, 12, 13, 15 (see underline)			Satisfaction with life (SWLS)^★★^
ADRQL: **Response to surroundings**	**p = 0.001**	0.168	Challenging behavior (CMAI)^★★★^
Non-significant independent variable: 2, 3, 4, 6, 7, 8, 9, 12, 13, 14 (see underline)			Responsible nurse (yes/no)^★^
			Circumstances of ratings^★★★^
			Time being on the ward (nurse)^★★^
			Days worked in advance of the ratings^★^

1: min. 1 challenging behavior (CMAI); 2: severity of dementia (GDS); 3: sex (residents); 4: sex (nurses); 5: responsible nurse (yes/no); 6: registered nurse (yes/no); 7: Burnout – emotional exhaustion (MBI-EE); 8: Burnout – personal accomplishment (MBI-PA); 9: Burnout – depersonalization (MBI-D); 10: Circumstances of ratings; 11: time being on the ward (nurse); 12: attitude – ADQ (total score); 13: empathy (JSPE); 14: satisfaction with life (SWLS); 15: days worked in advanced of the ratings^★^p < 0.05, ^★★^p < 0.10, ^★★★^p < 0.001.

**Table 4:** **Parameter estimates of factors associated with ADRQL-ratings**

	**ADRQL: Total score**	
	**β**	**95% CI**
No challenging behavior (CMAI)*	5.287	0.333; 10.185★
GDS ≤ 4***	11.005	-2.464; 24.473
GDS = 5***	11.663	3.966; 19.359★★
GDS = 6***	8.017	3.171; 12.863★★
MBI-EE low burden**	-8.266	-16.941; 0.408
MBI-EE moderate burden**	-10.536	-17.014; -4.058★★
Ratings while leisure time****	-1.152	-8.414; 6.110
Ratings in advance of a shift****	3.504	-4.919; 11.927
Ratings while the shift****	-10.099	-16.488; -3.709★★
Time being on the ward (nurse)^#^	3.173	1.290; 5.056★★
SWLS (nurse)^#^	1.366	0.679; 2.052★★★
	**ADRQL: Social interaction**	
	**β**	**95% CI**
GDS ≤ 4***	19.863	2.219; 37.508★
GDS = 5***	7.953	-2.130; 18.035
GDS = 6***	6.861	0.513; 13.210★
Nursing assistance******	9.164	-3.126; 9.735★
Ratings while leisure time****	-7.524	-17.038; 1.989
Ratings in advance of a shift****	3.409	-7.626; 14.444
Ratings while the shift****	-12.582	-20.953; -4.211★★
SWLS (nurse)^#^	0.929	0.030; 1.828★
	**ADRQL: Awareness of self**	
	**β**	**95% CI**
GDS ≤ 4***	22.438	4.826; 40.051★
GDS = 5***	17.545	7.481; 27.609★★
GDS = 6***	7.206	0.869; 13.543★
Ratings while leisure time****	9.938	0.442; 19.435★
Ratings in advance of a shift****	2.119	-8.896; 13.135
Ratings while the shift****	-4.609	-12.965; 3.747
SWLS (nurse)^#^	1.193	0.295; 2.090★★
Days worked in advance of the ratings^#^	1.277	0.095; 2.460★
	**ADRQL: Moods and feelings**	
	**β**	**95% CI**
No challenging behavior (CMAI)*	8.040	1.920; 14.160★★
GDS ≤ 4***	5.732	-11.096; 22.561
GDS = 5***	9.020	-0.596; 18.636
GDS = 6***	8.376	2.321; 14.341★★
MBI-EE low burden**	-12.872	-23.710; -2.033★
MBI-EE moderate burden**	-14.062	-22.156; -5.968★★
MBI-PA low burden**	-1.331	-10.978; 8.317
MBI-PA moderate burden**	-11.463	-21.939; -0.988★
Ratings while leisure time****	-1.559	-10.633; 7.515
Ratings in advance of a shift****	2.310	-8.215; 12.835
Ratings while the shift****	-12.954	-20.938; -4.971★★
Time being on the ward (nurse)^#^	5.320	2.967; 7.673★★★
SWLS (nurse)^#^	1.537	0.680; 2.395★★★
	**ADRQL: Enjoyment of activities**	
	**β**	**95% CI**
GDS ≤ 4***	14.401	-9.406; 38.207
GDS = 5***	22.707	9.104; 36.311★★
GDS = 6***	15.446	6.881; 24.012★★★
SWLS (nurse)^#^	1.676	0.463; 2.889★★
	**ADRQL: Response to surroundings**	
	**β**	**95% CI**
No challenging behavior (CMAI)*	12.639	5.622; 19.655★★★
Being no responsible nurse*****	7.225	0.140; 14.309★
Ratings while leisure time****	-4.483	-14.435; 5.470
Ratings in advance of a shift****	2.875	-8.885; 14.634
Ratings while the shift****	-18.804	-28.730; -8.879★★★
Time being on the ward (nurse)^#^	4.384	1.341; 7.427★★
Days worked in advance of the ratings^#^	-1.381	-2.664; -0.098★★

*as compared to min. 1 challenging behavior; **as compared to high burden in this scale; ***as compared to GDS 7; ****as compared to ratings after the shift; *****as compared to being responsible nurse; ******as compared to Registered Nurses; ^#^co-variable; ★p < 0.05, ★★p < 0.10, ★★★p < 0.001.

CMAI: Cohen-Mansfield Agitation Inventory; MBI-EE: Maslach Burnout Inventory – Emotional Exhaustion; MBI-PA: Maslach Burnout Inventory – Personal Accomplishment; SWLS: Satisfaction with Life Scale; GDS: Global Deterioration Scale; CI: Confidence Interval.

### Associated factors of the QUALIDEM

Five out of the nine subscales and the total score of the QUALIDEM could significantly be explained by covariance analyses (Table 5). The explained proportion of variance is again up 53.2% (R^2^ = 0.532). Five subscales (‘total score’, ‘care relationship’, ‘positive affect’, ‘restless tense behaviour’, and ‘social isolation’) result in higher QoL in the case of absence of challenging behaviours or a higher burden for nurses (MBI) or nurses’ higher satisfaction with life (SWLS). Ratings during the shift are associated with lower QoL in three QUALIDEM subscales (‘positive affect’, ‘negative affect’, and ‘restless tense behaviour’). Two subscales (‘care relationship’ and ‘restless tense behaviour’) result in higher QoL in the case of lower severity of dementia (See Table 6).

**Table 5:** **Factors associated with QUALIDEM-ratings**

**Dependent variable**	**p-value**	**R** ^**2**^	**Significant independent variable**
QUALIDEM: Total score	p < 0.001	0.165	Challenging behavior (CMAI) ^★★★^
non-significant independent variable: 2, 3, 4, 5, 6, 7, 8, 10, 12, 13, 15 (see underline)			Burnout (MBI – D) ^★^
			Time being on the ward (nurse) ^★^
			Satisfaction with life (SWLS) ^★★^
QUALIDEM: Care relationship	p < 0.001	0.227	Challenging behavior (CMAI) ^★★★^
non-significant independent variable: 3, 4, 5, 6, 7, 8, 10, 11, 12, 13, 15 (see underline)			Severity of dementia (GDS) ^★^
			Burnout (MBI – D) ^★★^
			Satisfaction with life (SWLS) ^★★^
QUALIDEM: Positive affect	p < 0.001	0.182	Challenging behavior (CMAI) ^★★^
non-significant independent variable: 2, 3, 4, 5, 6, 7, 9, 11, 12, 13, 14, 15 (see underline)			Burnout (MBI-PA) ^★★^
			Circumstances of ratings^★★^
QUALIDEM Negative affect	p < 0.001	0.209	Sex (residents) ^★★^
non-significant independent variable: 1, 2, 4, 5, 6, 8, 11, 15 (see underline)			Burnout (MBI – EE) ^★★★^
			Burnout (MBI – D) ^★^
			Circumstances of ratings^★^
			Attitude (ADQ) ^★★^
			Empathy (JSPE) ^★^
			Satisfaction with life (SWLS) ^★★^
QUALIDEM Restless tense behavior	p < 0.001	0.244	Challenging behavior (CMAI) ^★★★^
non-significant independent variable: 3, 4, 6, 8, 12, 13, 15 (see underline)			Severity of dementia (GDS) ^★★★^
			Responsible nurse (yes/no) ^★^
			Burnout (MBI – EE) ^★^
			Burnout (MBI – D) ^★^
			Circumstances of ratings^★★^
			Time being on the ward (nurse) ^★^
			Satisfaction with life (SWLS) ^★★^
QUALIDEM Positive self image*	p = 0.033	0.486	---
non-significant independent variable: 1, 2, 3, 4, 5, 6, 7, 8, 9, 10, 11, 12, 13, 14, 15 (see underline)			
QUALIDEM Social relations	p = 0.088	0.099	Satisfaction with life (SWLS) ^★^
non-significant independent variable: 1, 2, 3, 4, 5, 6, 7, 8, 9, 10, 11, 12, 13, 15 (see underline)			
QUALIDEM Social isolation	p < 0.001	0.173	Challenging behavior (CMAI) ^★★★^
non-significant independent variable: 2, 3, 4, 5, 6, 7, 8, 9, 10, 11, 12, 13, 15 (see underline)			Satisfaction with life (SWLS) ^★★^
QUALIDEM Feeling at home*	p = 0.010	0.532	---
non-significant independent variable: 1, 2, 3, 4, 5, 6, 7, 8, 9, 10, 11, 12, 13, 14, 15 (see underline)			
QUALIDEM Having something to do*	p = 0.759	0.268	---
non-significant independent variable: 1, 2, 3, 4, 5, 6, 7, 8, 9, 10, 11, 12, 13, 14, 15 (see underline)			

1: min. 1 challenging behavior (CMAI); 2: severity of dementia (GDS); 3: sex (residents); 4: sex (nurses); 5: responsible nurse (yes/no); 6: registered nurse (yes/no); 7: Burnout – emotional exhaustion (MBI-EE); 8: Burnout – personal accomplishment (MBI-PA); 9: Burnout – depersonalization (MBI-D); 10: Circumstances of ratings; 11: time being on the ward (nurse); 12: attitude – ADQ (total score); 13: empathy (JSPE); 14: satisfaction with life (SWLS); 15: days worked in advanced of the ratings; *not for GDS = 7 ^★^p < 0.05, ^★★^p < 0.10, ^★★★^p < 0.001.

**Table 6:** **Parameter estimates of factors associated with QUALIDEM-ratings**

	**QUALIDEM total score**	
	**β**	**95% CI**
No challenging behavior (CMAI)*	11.482	5.104; 17.859★★★
MBI-D low burden**	-1.743	-20.541; 17.055
MBI-D moderate burden**	-12.940	-29.927; 4.047
Time being on the ward (nurse)^#^	2.507	0.078; 4.937★
SWLS (nurse)^#^	1.423	0.533; 2.312★★
	**QUALIDEM: Care relationship**	
	**β**	**95% CI**
No challenging behavior (CMAI)*	17.570	10.993; 24.147★★★
GDS ≤ 4***	-27.722	-47.329; -8.114★★
GDS = 5***	-2.061	-12.080; 7.958
GDS = 6***	0.600	-6.329; 7.528
MBI-D low burden**	-5.552	-24.938; 13.834
MBI-D moderate burden**	-17.252	-34.771; 0.267
SWLS (nurse)^#^	1.380	0.463; 2.297★★
	**QUALIDEM: Positive affect**	
	**β**	**95% CI**
No challenging behavior (CMAI)*	8.913	2.251; 15.575★★
MBI-PA low burden**	10.020	-0.436; 20.475
MBI-PA moderate burden**	17.057	5.713; 28.401★★
Ratings while leisure time****	8.737	-0.985; 18.460
Ratings in advance of a shift****	13.491	2.192; 24.789★★
Ratings while the shift****	-4.156	-12.763; 5.551
	**QUALIDEM: Negative affect**	
	**β**	**95% CI**
Male (residents)*****	12.852	5.140; 20.563★★
MBI-EE low burden**	-15.205	-29.352; -1.059★
MBI-EE moderate burden**	3.241	-7.285; 13.767
MBI-DP low burden**	-14.903	-38.606, 8.800
MBI-D moderate burden**	-24.105	-45.525; -2.685★
Ratings while leisure time****	7.261	-4.475; 18.998
Ratings in advance of a shift****	22.714	9.975; 36.354★
Ratings while the shift****	6.779	-3.611; 17.169
ADQ^#^	-1.204	-1.989; -0.419★★
JSPE^#^	-0.852	-1.657; -0.046★
SWLS (nurse) ^#^	1.939	0.818; 3.061*★
	**QUALIDEM: Restless Tense Behavior**	
	**β**	**95% CI**
No challenging behavior (CMAI)*	19.979	11.059; 28.899★★★
GDS ≤ 4***	-6.029	-32.621; 20.562
GDS = 5***	20.485	6.898; 34.074★★
GDS = 6***	15.116	5.720; 24.513★★
Being no responsible nurse******	9.630	0.723; 18.536★
MBI-EE low burden**	-20.363	-36.055; -4.671★
MBI-EE moderate burden**	-10.023	-21.699; 1.653
MBI-D low burden**	-10.641	-36.933; 15.650
MBI-D moderate burden**	-21.552	-45.311; 2.208
Ratings while leisure time****	1.334	-4.693; 25.564
Ratings in advance of a shift****	10.436	-4.693; 25.564
Ratings while the shift****	-13.395	-24.920; -1.871★
Time being on the ward (nurse)^#^	3.720	0.322; 7.119★
SWLS (nurse)^#^	1.689	0.445; 2.933★★
	**QUALIDEM: Social Isolation**	
	**β**	**95% CI**
No challenging behavior (CMAI)*	14.972	8.035; 21.910★★★
SWLS (nurse)^#^	1.316	0.349; 2.283★★

*as compared to min. 1 challenging behavior; **as compared to high burden in this scale; ***as compared to GDS 7; ****as compared to ratings after the shift; *****as compared to female; ******as compared to being responsible nurse; ^#^co-variable; ★p < 0.05, ★★p < 0.10, ★★★p < 0.001.

CMAI: Cohen-Mansfield Agitation Inventory; MBI-DP: Maslach Burnout Inventory – Depersonalization; MBI-EE: Maslach Burnout Inventory – Emotional Exhaustion; MBI-PA: Maslach Burnout Inventory – Personal Accomplishment; MBI-D: Maslach Burnout Inventory – Depersonalization; SWLS: Satisfaction with Life Scale; GDS: Global Deterioration Scale; ADQ: Approach to Dementia Questionnaire; JSPE: Jefferson Scale of Physician Empathy; CI: Confidence Interval.

## Discussion

The present study aimed to identify possible associated factores of nurse rated QoL of PwD in German nursing homes. By identifying such associated factors, the performance of QoL proxy-ratings can be improved. Taking these associated factors into account, ratings are more reliable and most notably more comparable.

The mean age, gender distribution as well as severity of dementia indicates a typical German and international nursing home population [15,17,30]. In addition, characteristics (age, proportion of female, etc.) of the included nurses are comparable to those of the larger nursing exit study in various European countries [17,31]. Thus, we can conclude that we have no sample bias and the results of the study are valid from this point of view.

### Identification of associated factors of proxy-rated QoL

We found various factors associated the proxy-ratings. First, the resident related factors are discussed. The data analyses show, that 13 out of 16 QoL scores (subscales and total scores) were significantly influenced by the assumed factors. Residents’ sex only influences the subscale negative affect of the instrument QUALIDEM. This is largely in line with the results of a previous study, which concludes that QoL is independent from sex [5,32]. However, occurrence of challenging behaviour is associated with residents’ lower QoL in the present as well as in previous studies [5,32]. In addition, the severity of dementia effects the proxy-ratings. Lower severity is described to be associated with higher QoL, as it is in the present study. Due to the presence of challenging behaviour and that the level of severity of dementia may change over time [16], longitudinal studies have to consider these aspects as possible confounders in order to adjust analyses. If not accounted for, possible effects of care provision as well as interventional effects might be hidden and no clear conclusions could be drawn.

In addition to resident characteristics we found various nurse related characteristics that influence the proxy-rated QoL of nursing home residents with dementia. Due to the lack of similar studies, a critical discussion of identified associated factors is difficult, particularly, caregiver burnout as lower burden, frequently results in higher QoL-ratings. This might result from the fact that nurses with a higher burden expect the person with dementia to be burdened as well and therefore assume a low QoL. However, this issue needs to be clarified in future research projects. Generally, the literature has described that nurses in German institutional care settings frequently suffer from burnout [33]. This has to be taken into account, when using nurses as surrogates for residents in any study.

The circumstances of proxy-ratings are relevant associated factors as well. In various studies, the time at which proxy-ratings are completed is not described. Based on the present results, different circumstances might lead to different ratings, which are also clinically relevant (β up to 18.8).

### Limitations

The study was performed as a single centre study using a convenience sample consisting of residents and nurses from Berlin, Germany. Although we do not believe we had a sampling bias, this may influence the results. In addition, about 14% of nurses did not participate in our study. This may limit our results. The achieved explanation of variance throughout the co-variance analyses is not very high, thus further associated factors might exist in addition to those identified in the present study, e.g., functional abilities or depression of residents. A larger sample with a more comprehensive profile of nurses may address this issue.

## Conclusion

Assessing QoL of PwD is acknowledged as a central component of health care and health care research. In later stages of dementia, proxy-reported information obtained from quality of life questionnaires is and will continue to be essential. However, methodological issues that underline this research - matters of measurement and instrument validity - have to receive more attention. Associated factors in proxy-ratings have to be routinely assessed in order to get more valid and comparable estimates. Especially in longitudinal studies, changes in QoL over time can be influenced by factors other than the primary goals e.g., the evaluation of an intervention.
